# The effects of different familial Alzheimer’s disease mutations on APP processing in vivo

**DOI:** 10.1186/s13195-017-0234-1

**Published:** 2017-02-16

**Authors:** Steinunn Thordardottir, Anne Kinhult Ståhlbom, Ove Almkvist, Håkan Thonberg, Maria Eriksdotter, Henrik Zetterberg, Kaj Blennow, Caroline Graff

**Affiliations:** 1 Department NVS, Division of Neurogeriatrics, Center for Alzheimer Disease Research, Karolinska Institutet, 141 57, Huddinge, Sweden; 2 Department of Geriatric Medicine, Karolinska University Hospital Huddinge, 141 86, Stockholm, Sweden; 3 Department NVS, Center for Alzheimer Research, Division of Translational Alzheimer Neurobiology, Karolinska Institutet, 141 57, Huddinge, Sweden; 4 Department NVS, Division for Clinical Geriatrics, Center for Alzheimer Disease Research, Karolinska Institutet, 141 57, Huddinge, Sweden; 5 Department of Psychiatry and Neurochemistry, Institute of Neuroscience and Physiology, The Sahlgrenska Academy at the University of Gothenburg, SE-431 80 Mölndal, Sweden; 6 UCL Insitute of Neurology, Queen Square, London, UK; 7 Clinical Neurochemistry Laboratory, Sahlgrenska University Hospital, Mölndal, SE-431 80 Mölndal, Sweden

**Keywords:** Alzheimer’s disease, Amyloid precursor protein, Biomarkers, Cerebrospinal fluid, Genetics

## Abstract

**Background:**

Disturbed amyloid precursor protein (APP) processing is considered to be central to the pathogenesis of Alzheimer’s disease (AD). The autosomal dominant form of the disease, familial AD (FAD), may serve as a model for the sporadic form of AD. In FAD the diagnosis of AD is reliable and presymptomatic individuals carrying FAD mutations can give valuable insights into the earliest stages of the disease where therapeutic interventions are thought to be the most effective.

**Methods:**

In the current cross-sectional study, products of APP processing (e.g., sAPPα, sAPPβ, Aβ_38_, Aβ_40_ and Aβ_42_) were measured in the cerebrospinal fluid (CSF) of individuals carrying one of three FAD mutations, *APPswe* (p.KM670/671NL), *APParc* (p.E693G) and *PSEN1* (p.H163Y), as well as in non-mutation carriers from the same families.

**Results:**

We observed pathological APP processing in presymptomatic carriers of FAD mutations, with different profiles of APP and Aβ isoforms in the three mutation carrier groups, *APPswe* (p.KM670/671NL), *APParc* (p.E693G) and *PSEN1* (p.H163Y), except for the well-established decrease in CSF Aβ_42_ that was found with all mutations.

**Conclusions:**

These findings add to the current evidence that AD pathophysiology differs between disease-causing mutations and can be monitored in the presymptomatic disease stage by CSF analyses. This may also be important from a therapeutic standpoint, by opening a window to monitor effects of disease-modifying drugs on AD pathophysiology.

## Background

Alzheimer’s disease (AD) is a progressive neurodegenerative disorder with pathological hallmarks in the brain, including extracellular amyloid-beta (Aβ) plaques and intracellular tangles of hyperphosphorylated tau protein. According to the amyloid cascade hypothesis, accumulation of Aβ in the brain is the driver of the disease process [1]. Aβ isoforms ending at amino acid 42 (Aβ_42_) are the most prone to aggregate, both into synaptotoxic oligomers and into fibrils, which are the main components of neuritic plaques [2]. Aβ is a product of the cleavage of the amyloid precursor protein (APP) by the proteases β-secretase (BACE1) and γ-secretase. Following APP proteolysis at the cell surface, Aβ and sAPPβ, are shed into the brain interstitial fluid, which communicates freely with the cerebrospinal fluid (CSF). APP can also be cleaved within its Aβ sequence by α-secretase in a nonamyloidogenic pathway, releasing a different N-terminal APP fragment, sAPPα [3].

The processing of APP is interesting from a therapeutic standpoint because modulation/inhibition at different stages can be feasible to direct APP processing towards the nonamyloidogenic pathway. Measuring the products of APP processing in CSF can clarify how the processing differs in vivo in individuals with AD pathology, compared with healthy controls. This is of particular interest in patients who are still in the preclinical stage of AD, where disease-modifying drug candidates are most likely to be successful.

**Fig. 1:**
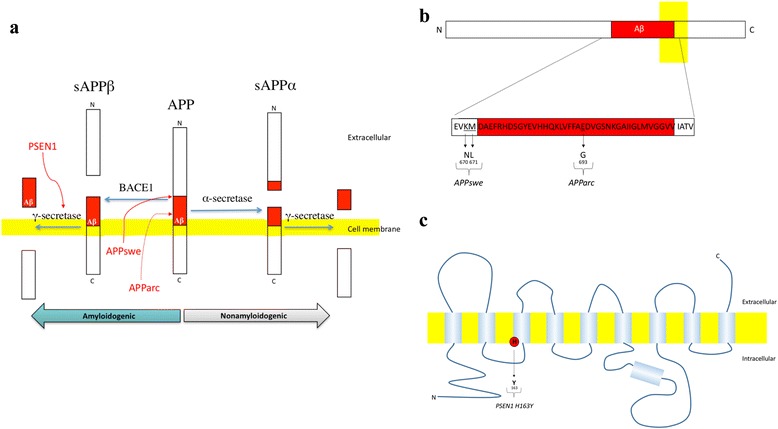
Effects of the *APPswe*, *APParc* and *PSEN1 H163Y* mutations on APP processing in vitro. **a** *APPswe* mutation is located in the extracellular domain of APP, at the BACE1 cleavage site, causing a 5–10-fold increase in the production of Aβ_40_ and Aβ_42_ [34]. Presenilin 1 is a subunit of the γ-secretase and most *PSEN1* mutations modulate the γ-secretase cleavage site-preference in a disease-promoting manner by inhibiting cleavages at Gly37, Gly38 and Val39 in the Aβ sequence, without affecting the production of Aβ_42_ and Aβ_40_ significantly [35]. Finally, the arctic APP mutation, located within the Aβ sequence, leads to the production of Aβ with higher propensity for protofibril formation than wild-type Aβ [6]. **b** More detailed overview of the position of the *APPswe* and *APParc* mutations within APP. *Letters*, amino acids; *numbers*, position of the amino acids within the APP sequence. **c** Schematic illustration of the transmembrane protein presenilin 1, a subunit of γ-secretase. The *PSEN1 H163Y* mutation is located in the third transmembrane domain of the protein. *APP* amyloid precursor protein

To learn more about APP and Aβ metabolism in familial AD (FAD), we measured sAPPα, sAPPβ, Aβ_38_, Aβ_40_ and Aβ_42_ in the CSF of individuals carrying autosomal dominant FAD-causing mutations, comparing them with noncarriers from the same families. Most of the subjects were still in the preclinical stage of the disease. We included carriers of three different mutations, *APPswe* [4, 5], *APParc* [6] and *PSEN1 H163Y* [7, 8], each of which causes distinct changes in APP processing in vitro. The effects of the different FAD mutations are illustrated in Fig. 1.

Aβ_42_ is a well-established biomarker, which is decreased in the CSF of individuals with AD, most probably due to the accumulation of Aβ_42_ in the brain [9]. The same does not apply to CSF Aβ_40_, which has been shown to be unchanged in AD [10–12]. Previous studies have shown conflicting results regarding the levels of other APP processing products in AD. CSF Aβ_38_ has been reported unchanged or increased in sporadic AD (SAD) [13, 14] but decreased in FAD [15, 16]. Aβ_16_ has been shown to be increased both in SAD and FAD [15, 16]. The levels of sAPPα and sAPPβ have been reported to be similar in FAD, SAD and controls [17, 18], but an increase in sAPPβ has been observed when comparing SAD with frontotemporal dementia [19]. When patients with SAD were compared with patients assessed as having other dementias than AD, the SAD patients had increased levels of both sAPPα and sAPPβ [20]. In contrast, one study reported a decrease in sAPPα in carriers of the *APPswe* mutation [21] and another a decrease both in sAPPα and sAPPβ in patients with advanced AD compared with healthy controls [10].

Here we speculated that the levels of the products of APP processing would already be abnormal in preclinical FAD and differ depending on the underlying mutation: that the carriers of the *APPswe* mutation would have increased levels of sAPPβ, Aβ_38_ and Aβ_40_ as well as decreased levels of sAPPα due to the increased affinity of BACE1 to the mutated APP sequence; that the *APParc* mutation carriers would have normal sAPPα and sAPPβ levels but decreased levels of Aβ_42_, and potentially Aβ_40_ due to fibril formation; and that subjects with the *PSEN1 H163Y* mutation would also have normal levels of sAPPα and sAPPβ, but decreased levels of Aβ_38_ as the mutated γ-secretase preferentially produces Aβ_40_ and Aβ_42._

In the therapeutic sense it was interesting to see whether these hypotheses would be correct or whether we would find evidence of a common pathological aspect of APP processing in carriers of the different FAD mutations.

Finally, we calculated the sAPPβ/sAPPα ratio and the Aβ_42_/Aβ_40_ ratio, both of which are of interest as potential biomarkers.

## Methods

### Study population

Members of three Swedish families segregating three known mutations leading to autosomal dominant AD, *APPswe* (p.KM670/671NL), *APParc* (p.E693G) and *PSEN1* (p.H163Y), were included in the study. The average age at onset of the first clinically relevant symptoms for each family is: 54 ± 4 years for *APPswe* (based on 19 affected cases), 56 ± 4 years for *APParc* (based on 12 affected cases) and 51 ± 7 years for *PSEN1 H163Y* (based on 11 affected cases). The average age at onset in each family is normally distributed and is therefore presented as a mean with standard deviation. Participants were recruited to a longitudinal clinical and experimental FAD study through the Genetics Unit, which provides genetic counseling at the Memory Clinic at the Karolinska University Hospital, and were examined between 1993 and 2011. The FAD study is a prospective study including a thorough clinical evaluation, a comprehensive neuropsychological test battery, neuroimaging (3Tesla MRI), electroencephalography and biochemical assessments, including collection of CSF. For AD diagnosis, the criteria for dementia according to the National Institute of Neurological Disorders and Stroke, Washington DC, and Alzheimer Disease and Related Disorders Association, Washington DC (NINCDS-ADRDA) were used [22]; and for the diagnosis of mild cognitive impairment (MCI) we used the criteria of the International Working Group on Mild Cognitive Impairment [23]. In a person with MCI the ability to perform activities of daily living is preserved, while at the same time there is evidence of cognitive decline either objectively measured over time or subjectively reported by the individual/an informant in conjunction with objective deficits. The study subjects are a subsample of individuals at 50% risk of carrying one of the mutations leading to FAD who gave informed written consent to and underwent lumbar puncture (*n* = 36). The CSF was collected at baseline/the entrance of each participant into the study, during the time period of 1993–2011, and the age of the participants presented in the following sections is the age at CSF collection. Of the 36 subjects who underwent the procedure, 18 came from a family segregating the *APPswe* mutation, 10 from a family segregating the *APParc* mutation and eight from a family segregating the *PSEN1 H163Y* mutation. All of the subjects received genetic counseling in conjunction with their participation in the study. The participants, clinicians and researchers involved in the study were blind to the mutation status of the asymptomatic participants unless the participant opted for presymptomatic genetic testing (in a clinical genetics setting). All study procedures were approved by the Regional Ethical Review Board in Stockholm, Sweden.

### Cerebrospinal fluid sampling

CSF samples were collected by lumbar puncture in the L3/L4 or L4/L5 interspace at variable time points during the day. According to current recommendations on CSF sampling and handling, diurnal variation of AD biomarkers should not be a concern [24]. Some of the participants received premedication with 5 mg diazepam and 1 g paracetamol prior to the procedure. Immediately after collection the CSF was centrifuged at 2200 × *g* at room temperature for 10 minutes. The supernatant was collected and aliquoted into polypropylene cryotubes. The aliquots used in the current study were stored at −80 °C and had been thawed and refrozen once before being thawed for analysis in this study.

### Assay of sAPPα, sAPPβ, Aβ_38_, Aβ_40_ and Aβ_42_

The CSF samples were all analyzed at the same time by electrochemiluminescence technology (Meso Scale Discovery, Gaithersburg, MD, USA). An analysis of the stability of the frozen CSF samples had been performed previously and did not raise concerns. Concentrations of Aβ_38_, Aβ_40_ and Aβ_42_ were analyzed using the MS6000 Human Abeta 3-Plex Ultra-Sensitive Kit (the 6E10 version), while β-secretase cleaved soluble APP (sAPP-β) and α-secretase cleaved soluble APP (sAPP-α) in CSF were analyzed using the MS6000 Human sAPPalpha/sAPPbeta Kit, following the recommendations by the manufacturer, and as described previously [25]. Because the *APPswe* mutation changes the neo-epitope recognized by the capturing antibody in the sAPPβ assay, CSF sAPPβ levels in carriers of the *APPswe* mutation were not included in the study.

### Genetic analysis

#### Apolipoprotein E

The *APOE* genotyping was performed for SNPs rs7412 and rs429358 using TaqMan® SNP Genotyping Assays (ABI, Foster City, CA, USA) according to the manufacturer’s protocol. The amplified products were run on 7500 fast Real-Time PCR Systems (ABI).

#### Mutation analyses in APP and PSEN1

Exons 16 and 17 in *APP* were sequenced to screen for the KM670/671NL and the E693G mutations [12, 14]. To confirm the H163Y mutation in *PSEN1*, exon 6 was sequenced [26]. DNA was amplified using AmpliTaq Gold® 360 PCR Master Mix (Applied Biosystems, Foster City, CA, USA). Primer sequences and PCR conditions are available upon request. The Big Dye® terminator v3.1 Cycle sequencing Kit (Applied Biosystems, Austin, TX, USA) was used for Sanger sequencing. The exons in *APP* and *PSEN1* were sequenced in both directions and analyzed on an ABI3500 Genetic Analyzer (Applied Biosystems, Foster City).

### Statistical analysis

Because of the fact that the age at symptom onset in FAD varies between families, each subject’s age relative to the mean age at clinical onset in their respective family was calculated. Unpaired *t* tests and Pearson correlations were applied to normally distributed data, while the Mann–Whitney U test and Spearman correlations were performed on data that was not normally distributed. The distribution of data was assessed with the D’Agostino–Pearson normality test. Thus age, years to predicted family specific clinical onset and CSF biomarker levels were compared between noncarriers of the FAD mutations and the mutation carrier group as a whole as well as the noncarriers and carriers of each of the three mutations separately. Fisher’s exact test was used to compare gender and the frequency of the *APOE* ε4 allele between the noncarriers and each of the mutation carrier groups.

Exploratory Pearson/Spearman correlations were performed between biomarker levels, as well as between biomarker levels and predicted age at symptom onset. Two-tailed *p* < 0.05 was considered significant.

## Results

### Demographics of the study population

Of the 36 subjects who underwent a lumbar puncture, 19 were mutation carriers (MC) and 17 were noncarriers (NC). Of the MC, six carried a *PSEN1 H163Y* mutation, four carried an *APParc* mutation and nine carried an *APPswe* mutation (see Table 1 for a summary of the demographic data of the study population). There was no significant difference between the MC group as a whole, or any of the MC subgroups, and the NC regarding gender distribution, mean age or mean number of years to predicted family specific onset. For the sake of anonymity, the gender distribution in each mutation group is not revealed in Table 1, because this could cause certain participants to be able to deduct their mutation status from the data. The *PSEN1* carriers were somewhat younger than the NC, but this difference did not reach statistical significance. Furthermore, there was no significant difference in the prevalence of *APOE* ε4 carriers between the NC group and the different MC groups and there were only two individuals, a noncarrier and a carrier of the *APPswe* mutation, who were *APOE* ε4 homozygotes. At the time of CSF sampling, three of the *APPswe* carriers fulfilled the diagnostic criteria for AD with dementia, two *APPswe* carriers and one *PSEN1* carrier fulfilled the criteria for MCI and the other 30 subjects (13 MC and 17 NC) were free of symptoms.

**Table 1:** Demographics of the study population

	All MC (*n* = 19)	*APPswe* (*n* = 9)	*APParc* (*n* = 4)	*PSEN1* (*n* = 6)	NC (*n* = 17)
Age (SD)	46.7 (11.9)	51.2 (12.1)	50 (6.6)	33.7 (7.0)	47.8 (10.2)
Years to onset (SD)	−7.7 (12.3)	−1.8 (12.1)	−7 (6.6)	−21.3 (7.0)	−6.9 (9.9)
*ApoE* ε4 carrier	9/19	5/9	1/4	3/6	7/17
Normal cognition	13/19	4/9	4/4	5/6	17/17
MCI	3	2	-	1	-
AD	3	3	-	-	-

*AD* Alzheimer’s disease, *MC* mutation carriers, *MCI* mild cognitive impairment, *NC* noncarriers

### Levels of sAPPα, sAPPβ, Aβ_38_, Aβ_40_ and Aβ_42_

**Fig. 2:**
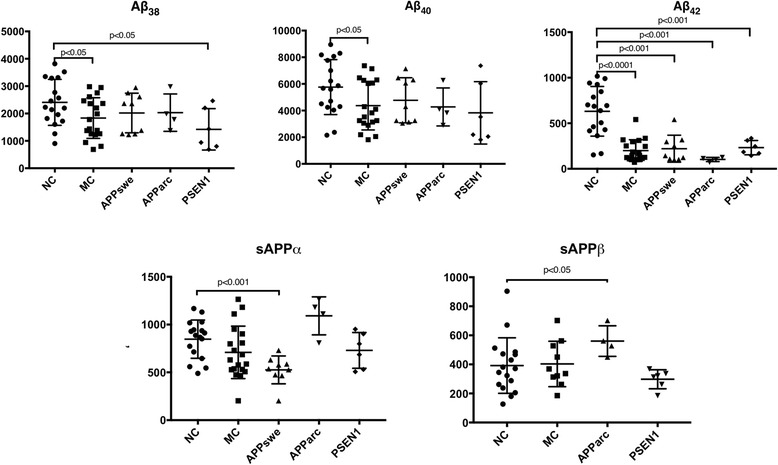
CSF levels of sAPPα, sAPPβ, Aβ_38_, Aβ_40_ and Aβ_42_ in carriers of three FAD mutations. Scatter plots showing levels of sAPPα, sAPPβ, Aβ_38_, Aβ_40_ and Aβ_42_ in carriers of three different mutations leading to FAD compared with healthy family members (NC). The mutation carriers were compared with the NC as one group (MC) and by type of mutation (*APPswe*, *APParc* and *PSEN1*). Only carriers of the *APParc* and *PSEN1* mutations are included in the calculation of the mean level of sAPPβ. The biomarker levels in the NC group were normally distributed, except for sAPPβ. Thus, the Mann–Whitney U test was used when comparing sAPPβ between groups. Also, the Mann–Whitney U test was used when comparing the *APParc* and *PSEN1* carriers with the NC due to the small sample sizes of these two MC groups. The unpaired *t* test was used for the rest of the data, except when comparing Aβ_42_ between the NC and MC and comparing sAPPα between the NC and *APPswe* (here we used the Mann–Whitney *U* test because these data were not normally distributed). *Aβ* amyloid beta, *MC* mutation carriers, *NC* noncarriers

There was no significant difference in the levels of sAPPα when comparing the MC group as a whole with the NC. The same applied to sAPPβ when the *PSEN1* and *APParc* carriers were grouped together and compared with the NC. In contrast, all of the measured Aβ isoforms, Aβ_38_, Aβ_40_ and Aβ_42_, were significantly lower in the MC group. When the MC were grouped by their specific mutation, a somewhat different pattern emerged. The carriers of the *APPswe* mutation had significantly lower levels of sAPPα than NC (note that *APPswe* MC were not included in the analysis of CSF sAPPβ because the end-specific capture antibody in the assay will not react with sAPPβ modified at positions 670/671 by the mutation), normal levels of Aβ_38_ and Aβ_40_ and significantly decreased levels of Aβ_42_. The *APParc* carriers shared the same pattern of the Aβ isoforms as the *APPswe* carriers, but had normal levels of sAPPα and high sAPPβ levels. Finally, the *PSEN1* carriers had low levels of both Aβ_38_ and Aβ_42_, and normal levels of Aβ_40_, sAPPα and sAPPβ. See Fig. 2 for a summary of the levels of the APP processing products.

These calculations were repeated after excluding all subjects with MCI or AD diagnosis. This did not change the results for the levels of sAPPα, sAPPβ and Aβ_42_, with all of the significant differences in Fig. 2 remaining significant. The levels of Aβ_38_ were no longer significantly lower in the MC group as a whole when subjects with MCI and AD had been excluded, but remained significantly low in the *PSEN1* carrier group. The results for Aβ_40_ remained unchanged after removing MCI and AD subjects, except the Aβ_40_ levels of the *PSEN1* carriers, which were significantly lower than in the NC when the *PSEN1* carrier with MCI diagnosis was excluded.

### sAPPβ/sAPPα ratio and Aβ_42_/Aβ_40_ ratio

There was no significant difference in the sAPPβ*/*sAPPα ratio when comparing the *PSEN1* and *APParc* carriers with the NC, irrespective of whether the NC were compared with the MC as a whole or with a subgroup with a specific mutation. The same applied when the NC were compared with the presymptomatic *PSEN1* carriers only (data not shown).

The Aβ_42_/Aβ_40_ ratio was significantly lower in the MC group as a whole than in the NC group (0.05 vs 0.11, *p* < 0.0001). The same applied to all the subgroups, the *APPswe* carriers vs the NC (0.04 vs 0.11 *p* < 0.0001), the presymptomatic *APPswe* carriers vs the NC (0.05 vs 0.11, *p* = 0.002), the *APParc* carriers vs the NC (0.03 vs 0.11, *p* < 0.001), the *PSEN1* carriers vs the NC (0.07 vs 0.11, *p* < 0.01) and the presymptomatic *PSEN1* carriers vs the NC (0.07 vs 0.11, *p* < 0.01).

### Correlations between the different products of APP processing

Because of the small size of the *APParc* mutation carrier group (*n* = 4) no correlation calculations were performed involving that group alone. As has been reported previously [27], there was a positive correlation between sAPPα and sAPPβ in the MC group as a whole (excluding the *APPswe* carriers as explained earlier), as well as in the NC group. When looking only at the *PSEN1* carriers, this correlation failed to reach significance, probably due to the small number of individuals in the group.

A strong positive correlation was observed between Aβ_38_ and Aβ_40_ in all groups. There was a significant correlation between Aβ_40_ and Aβ_42_ as well, in all groups except the *PSEN1* carriers. Interestingly, there was a positive correlation between sAPPα and all the measured Aβ isoforms in the NC group, but not in any of the mutation carrier groups. Also, the NC group was the only group showing a significant positive correlation between Aβ_38_ and Aβ_42_.

The correlations between sAPPα, sAPPβ, Aβ_38_, Aβ_40_ and Aβ_42_ are summarized in Table 2.

**Table 2:** Correlations between the products of APP processing in FAD mutation carriers

	CSF biomarker	sAPPα	sAPPβ	Aβ_38_	Aβ_40_	Aβ_42_
MC	sAPPα		*0.87 (<0.01)*	0.2 (0.4)	0.09 (0.7)	-0.2 (0.4)
	sAPPβ	*0.87 (<0.01)*		0.2 (0.3)	0.1 (0.6)	-0.2 (0.5)
	Aβ_38_	0.2 (0.4)	0.2 (0.3)		*0.93 (<0.0001)*	0.44 (0.06)
	Aβ_40_	0.09 (0.7)	0.1 (0.6)	*0.93 (<0.0001)*		*0.6 (0.01)*
	Aβ_42_	-0.2 (0.4)	-0.2 (0.5)	0.44 (0.06)	*0.6 (0.01)*	
*APPswe*	sAPPα			0.2 (0.6)	0.2 (0.7)	-0.03 (0.9)
	sAPPβ	n.a.	n.a.	n.a.	n.a.	n.a.
	Aβ_38_	0.2 (0.6)			*0.9 (<0.001)*	0.7 (0.06)
	Aβ_40_	0.2 (0.7)		*0.9 (<0.001)*		*0.8 (0.01)*
	Aβ_42_	-0.03 (0.9)		0.7 (0.06)	*0.8 (0.01)*	
*PSEN1*	sAPPα		0.66 (0.18)	0.20 (0.7)	0.20 (0.7)	0.086 (0.9)
	sAPPβ	0.66 (0.18)		0.60 (0.2)	0.60 (0.2)	0.54 (0.3)
	Aβ_38_	0.20 (0.7)	0.60 (0.2)		*1 (<0.01)*	0.83 (0.06)
	Aβ_40_	0.20 (0.7)	0.60 (0.2)	*1 (<0.01)*		0.83 (0.06)
	Aβ_42_	0.086 (0.9)	0.54 (0.3)	0.83 (0.06)	0.83 (0.06)	
NC	sAPPα		*0.64 (<0.01)*	*0.71 (<0.01)*	*0.72 (<0.01)*	*0.65 (<0.01)*
	sAPPβ	*0.64 (<0.01)*		0.28 (0.3)	0.33 (0.2)	0.29 (0.3)
	Aβ_38_	*0.71 (<0.01)*	0.28 (0.3)		*0.98 (<0.0001)*	*0.95 (<0.0001)*
	Aβ_40_	*0.72 (<0.01)*	0.33 (0.2)	*0.98 (<0.0001)*		*0.98 (<0.0001)*
	Aβ_42_	*0.65 (<0.01)*	0.29 (0.3)	*0.95 (<0.0001)*	*0.98 (<0.0001)*	

Correlations between the products of APP processing in the mutation carrier group as a whole (MC), carriers of the *APPswe* and *PSEN1* mutations separately and in the nonmutation carriers (NC)

Numbers represent Pearson/Spearman correlation coefficients (*p* values) (significant correlations in italics)

*APP* amyloid precursor protein, *FAD* familial Alzheimer’s disease, *Aβ* amyloid beta, *MC* mutation carriers, *NC* noncarriers

### Correlations between sAPPα, sAPPβ, Aβ_38_, Aβ_40_ and Aβ_42_ and years to expected symptom onset

**Fig. 3:**
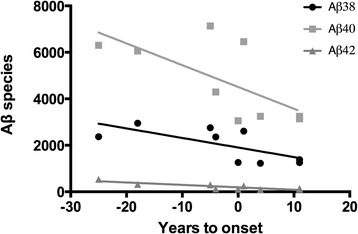
*APPswe*: correlations between years to onset and Aβ species. Pearson correlations between the Aβ isoforms Aβ_38_, Aβ_40_ and Aβ_42_ and years to expected onset of the first clinically relevant symptoms of FAD in carriers of the *APPswe* mutation. *X* axis, −10 represents an individual with 10 years left to the expected onset (which is set to 0) while 10 represents an individual 10 years past the expected onset. All three correlations were significant, Aβ_38_ (*r* = −0.69, *p* = 0.04), Aβ_40_ (*r* = −0.67, *p* = 0.05) and Aβ_42_ (*r* = −0.86, *p* < 0.01), decreasing in subjects sampled closer to the expected onset. *Aβ* amyloid beta

Correlations between sAPPα, sAPPβ, Aβ_38_, Aβ_40_, Aβ_42_ and years to expected symptom onset were calculated to see whether the levels of these markers changed as the onset of symptoms approached. No such changes were observed in any of the five markers in the whole MC group, the *PSEN1* group or in the NC group. In the *APPswe* mutation carriers, the concentration of Aβ_38_ (*r* = −0.69, *p* = 0.04), Aβ_40_ (*r* = −0.67, *p* = 0.05) and Aβ_42_ (*r* = −0.86, *p* < 0.01) decreased significantly when the expected symptom onset approached and beyond (see Fig. 3). When subjects with a dementia diagnosis were excluded from the *APPswe* mutation carrier group these correlations failed to reach significance, apart from Aβ_42,_ which still showed a decrease as age at onset approached (*r* = −0.85, *p* = 0.03). It was not possible to repeat this calculation with only presymptomatic *APPswe* mutation carriers because their number is too small.

### Correction for multiple comparisons

All of the significant correlations presented in Table 2 remained significant after correcting for multiple comparisons using the false discovery rate (FDR) correction. The same applied to the biomarker levels presented in Fig. 2, except for the low Aβ_38_ levels in the MC and the *PSEN1* groups and the low Aβ_40_ levels in the MC group which did not survive this correction.

## Discussion

Compared with noncarriers, carriers of the *APPswe* mutation have significantly decreased CSF levels of sAPPα and Aβ_42_ whereas the levels of Aβ_38_ and Aβ_40_ were not significantly different. The decreased levels of Aβ_42_ are expected, because Aβ_42_ is a well-established AD biomarker and has been shown to be decreased in AD patients in numerous studies [9]. The sAPPα levels have also been shown to be reduced in a previous study on some of the same individuals [21]. This could be due to the increased activity of the amyloidogenic pathway of APP processing in *APPswe* carriers at the expense of the nonamyloidogenic pathway in which sAPPα is produced.

In the *PSEN1* carriers the CSF levels of sAPPα and sAPPβ were unchanged, whereas sAPPβ was increased in the *APParc* carriers. The *APParc* carriers also showed the expected decrease in Aβ_42_ levels, while both Aβ_38_ and Aβ_42_ were decreased in the *PSEN1* carriers. The decrease in Aβ_38_ has been observed in previous studies on *PSEN1* carriers [15, 16] and the same applies to the normal levels of sAPPα and sAPPβ in the *PSEN1* carriers [17, 18]. Amyloid plaques including Aβ_38_ deposits, have been shown to be limited to FAD cases and this accumulation of Aβ_38_ in the brain could explain the low levels Aβ_38_ in CSF [28]. Another, possibly coexisting, explanation could be that the mutated γ-secretase preferentially produces Aβ_40_ and Aβ_42_, at the expense of Aβ_38_. The decrease in Aβ_38_ levels seems quite robust, remaining significant when only presymptomatic *PSEN1* carriers were included. In contrast, the observed reduced levels of Aβ_38_ for the whole MC group were no longer significantly low when looking only at presymptomatic individuals. Taken together our results emphasize the differences in production and metabolism of Aβ isoforms in different FAD mutations. The results for the *PSEN1* and *APParc* carriers fit our hypothesis quite nicely, apart from the observed increased levels of sAPPβ in the *APParc* carriers. A possible explanation for this observation might be that increased fibril formation and lack of monomeric Aβ isoforms somehow stimulates amyloidogenic APP processing.

When looking at correlations between years to symptom onset and the different APP processing products it is interesting to see that the products remain stable in subjects at different time points from expected onset in the MC group as a whole and in the *PSEN1* carriers alone. The Aβ isoforms remained low in the MC compared with the NC, while there was no difference in the levels of sAPPα and sAPPβ in the MC and the NC. Very few studies have looked at APP processing in preclinical FAD [15–17] and to our knowledge none have correlated the APP processing products to years to symptom onset.

According to one study on SAD, there were no differences in BACE1 activity, sAPPα, sAPPβ and Aβ_40_ concentrations between AD patients and controls until the levels of all of these biomarkers dropped in advanced stages of AD [10]. The only mutation carrier group in the current study that showed a change in these biomarkers in relation to expected symptom onset was the *APPswe* group, in which the levels of all of the Aβ isoforms decreased with progression of the disease. This resonates quite well with the study by Rosen et al. [10] on SAD, because the *APPswe* mutation carriers were the only group including individuals that already had a dementia diagnosis. The correlation ceased to be significant when the subjects with dementia were excluded, apart from Aβ_42_, which still showed a decrease over time. A longitudinal study on the subjects included here is ongoing and may shed light on the changes in these markers over time through repeated sampling from the same individuals.

We replicated the previously observed positive correlation between sAPPα and sAPPβ in both the MC group (excluding the *APPswe* carriers) and the NC group. This observation correlates well with other studies including healthy individuals as well as subjects with MCI, FAD and SAD, suggesting that the α and β pathways are noncompetitive [17, 20, 26, 27, 29, 30]. This positive correlation also applies to the MC in this study, possibly excluding the *APPswe* carriers because they had low levels of sAPPα. Because of limitations in the sAPPβ assay we do not have information on the levels of sAPPβ in the *APPswe* carriers and therefore can only speculate on the correlation between sAPPα and sAPPβ in this group. It is interesting that all three Aβ isoforms are positively correlated to sAPPα but not to sAPPβ in the NC group, as sAPPα is not a product of the same APP cleaving pathway as Aβ. A possible explanation would be that all of the measured Aβ isoforms are more abundant in the CSF of healthy individuals with high levels of sAPPα, as the threshold for Aβ aggregation in the brain has not yet been reached. Aβ_38_ and Aβ_40_ have a positive correlation to each other and the same is true for Aβ_40_ and Aβ_42_. This suggests that the Aβ_42_ decrease in CSF in FAD may be accompanied by a decrease in Aβ_40_ and possibly in Aβ_38_ as well, although the current study failed to show a significant correlation between Aβ_42_ and Aβ_38_.

Finally, we did not observe a difference in the sAPPβ*/*sAPPα ratio between the MC and the NC. The sAPPβ*/*sAPPα ratio has been suggested as a biomarker for brain amyloidosis [30], but our findings do not support it as a marker of early pathology. This should be interpreted with caution, however, because the possibility remains that the results were negative due to the small sample size in this instance (only 10 MC from the *PSEN1* and *APParc* families because data on sAPPβ were not included from the *APPswe* MC). As expected, the Aβ_42_/Aβ_40_ ratio was low in the MC group as a whole and in the MC subgroups compared with the NC. This difference remained statistically significant when symptomatic individuals had been excluded, supporting its role as a robust early AD biomarker.

The method used in the current study to measure the potential biomarkers sAPPβ and sAPPα has recently been analytically validated using novel standard operating procedures developed within the BIOMARKAPD project [31]. This validation increases the credibility of the results obtained with this method and decreases the likelihood of analysis bias.

The results discussed were not corrected for multiple comparisons and the differences observed in Aβ_38_ and Aβ_40_ levels between groups did not survive a FDR correction. When interpreting the results of the FDR correction one should keep in mind that the study was hypothesis driven and the biomarkers carefully selected before any statistical analysis was performed. Therefore, the study design itself reduces the likelihood of false positives. Also, the small sample sizes, which often require nonparametric testing, already reduce the power to detect true differences between groups. The possibility still remains that the differences observed in Aβ_38_ and Aβ_40_ levels between groups were significant by chance, as suggested by the FDR correction. However, we feel that this possibility is not great enough to disregard the results altogether and that they warrant further study in larger cohorts.

A limitation to this study is the number of included subjects and this should be taken into account when interpreting the results. The current study, however, is larger than previous studies on other aspects of APP processing which have included FAD subjects and replicates some of their results, underlining the robustness of the data acquired from this population. Even though the small sample size might have had some effect on the outcomes presented here, they are at the very least strongly hypothesis generating. Another limitation is the cross-sectional design of the study, which makes it impossible to assess changes in APP processing products over time. This will be addressed in a longitudinal study on the same subjects, which is ongoing. The usefulness of FAD subjects as models for SAD has been supported by several studies [32], but the possibility still exists that these results might not be generalizable to SAD.

## Conclusion

We observed pathological APP processing in presymptomatic carriers of FAD mutations, which adds to the current evidence that AD pathogenesis is present before the development of clinical symptoms [33]. We found no apparent common aspect of APP processing in the three mutation carrier groups, apart from the well-known decrease in CSF Aβ_42_, which is an early event in AD pathogenesis. Perhaps this decrease is secondary to the aggregation of brain amyloid, rather than a result of the APP processing steps, and thus a poor marker for the primary etiology. If the same type of treatment was to be used in presymptomatic FAD mutation carriers regardless of the type of mutation, these results suggest that a treatment interfering with the final product of APP processing, Aβ, would be a feasible option.

## Abbreviations

AD: Alzheimer’s disease
APP: Amyloid precursor protein
CSF: Cerebrospinal fluid
FAD: Familial Alzheimer’s disease
FDR: False discovery rate
MC: Mutation carrier
MCI: Mild cognitive impairment
MRI: Magnetic resonance imaging
NC: Noncarrier
PCR: Polymerase chain reaction
SAD: Sporadic Alzheimer’s disease
SD: Standard deviation
